# Coupling Removal of P-Chloronitrobenzene and Its Reduction Products by Nano Iron Doped with Ni and FeOOH (nFe/Ni-FeOOH)

**DOI:** 10.3390/ma15051928

**Published:** 2022-03-04

**Authors:** Jing Liang, Zhixue Li, Emmanuella Anang, Hong Liu, Xianyuan Fan

**Affiliations:** 1 College of Environmental Engineering, Nanjing Polytechnic Institute, Nanjing 210048, China; liangjing@njpi.edu.cn; 2 College of Resource and Environmental Engineering, Wuhan University of Science and Technology, Wuhan 430081, China; zhixueli@wust.edu.cn (Z.L.); anangemmanuella@gmail.com (E.A.); fanxianyuan@wust.edu.cn (X.F.); 3 Hubei Key Laboratory for Efficient Utilization and Agglomeration of Metallurgic Mineral Resources, Wuhan University of Science and Technology, Wuhan 430081, China

**Keywords:** p-chloronitrobenzene, nano iron, Ni, FeOOH

## Abstract

The removal of chlorinated pollutants from water by nanoparticles is a hot topic in the field of environmental engineering. In this work, a novel technique that includes the coupling effect of n-Fe/Ni and its transformation products (FeOOH) on the removal of p-chloronitrobenzene (p-CNB) and its reduction products, p-chloroaniline (p-CAN) and aniline (AN), were investigated. X-ray diffraction (XRD) and transmission electron microscopy (TEM) were employed to characterize the nano-iron before and after the reaction. The results show that Fe^0^ is mainly oxidized into lath-like lepidocrocite (γ-FeOOH) and needle-like goethite (α-FeOOH) after 8 h of reaction. The coupling removal process and the mechanism are as follows: Fe^0^ provides electrons to reduce p-CNB to p-CAN and then dechlorinates p-CAN to AN under the catalysis of Ni. Meanwhile, Fe^0^ is oxidized to FeOOH by the dissolved oxygen and H_2_O. AN is then adsorbed by FeOOH. Finally, p-CNB, p-CAN, and AN were completely removed from the water. In the pH range between 3 and 7, p-CAN can be completely dechlorinated by n-Fe/Ni within 20 min, while AN can be nearly 100% adsorbed by FeOOH within 36 h. When the temperature ranges from 15 °C to 35 °C, the dechlorination rate of p-CAN and the removal rate of AN are less affected by temperature. This study provides guidance on the thorough remediation of water bodies polluted by chlorinated organics.

## 1. Introduction

P-chloronitrobenzene (p-CNB) has been classified as a persistent toxic substance in which the main pathway of release into the environment encompasses its utilization in the manufacture of dyes, corrosion inhibitors, medicines and pesticides [1,2]. The extent of its toxicity, carcinogenicity, mutagenicity and teratogenicity on humans and biodiversity has already been established [3,4,5,6,7]. Thus, it is very necessary to devise feasible and effective methods to remove p-CNB from the environment in order to prevent the fatal consequences of the chemical on human health and biodiversity. Researchers use both biological and chemical methods to remove p-CNB from the environment [5,8]. However, the biological method that included the use of an anaerobic sludge system to facilitate reduction of the p-CNB is not efficient due to its strong recalcitrance to biodegradation as well as its high biological toxicity [2,5]. The chemical methods, including photocatalytic oxidation [9], Fenton oxidation [10,11] and nanotechnology [2,12], have been widely used to reduce p-CNB. Among these chemical methods, nanotechnology (nanoscale zerovalent iron (Fe^0^)) has been identified as the most effective due to its relatively low cost, high adsorption capacity, strong reactivity and high reductivity [13,14,15,16,17].

As we all know, Fe^0^ is prone to passivation and agglomeration, which adversely affects its removal efficiency. In view of this, the iron-based bimetallic particles have been employed to solve the problem of passivation and agglomeration of Fe^0^, to accelerate corrosion and to potentially promote its reactivity through hydrogenation [18]. The synthesized bimetallic particles include doping Fe^0^ on transition metals such as Ni, Cu, Pt or Pd to form n-Fe/Ni, n-Fe/Cu, n-Fe/Pt or n-Fe/Pd [3]. However, applications of Pd and Pt are limited due to the high costs and Cu exhibits a weak promotion effect on the dechlorination of p-CNB by Fe^0^ [2]. By comparison, Ni as the doped metal of Fe^0^ displays high cost-effectiveness, along with the ability to form galvanic cells with Fe to promote the conversion of Fe to iron hydroxides [18].

In the groundwater/wastewater, the n-Fe/Ni reduces the p-CNB into p-chloroaniline (p-CAN) and aniline (AN), while the latter two products are environmental pollutants that significantly threaten humans and biodiversity [15]. Therefore, it incites the need to devise a more efficient technique to remove the p-CNB in its entirety.

In this study, a novel technique that includes the coupling effect of n-Fe/Ni and its transformation products (FeOOH) on the removal of p-CNB, p-CAN and AN is investigated. FeOOH has been reported to possess good adsorption properties for organics; therefore, it is often used to remove organic pollutants from water [19]. The n-Fe/Ni before and after the reaction were characterized by X-ray diffraction (XRD) and transmission electron microscopy (TEM). The effects of pH and temperature on the coupling removal of p-CNB, p-CAN and AN by n-Fe/Ni and FeOOH were investigated using batch experiments. The underlying process and mechanism of the removal of the p-CNB, p-CAN and AN by the n-Fe/Ni coupling with FeOOH were elucidated.

## 2. Materials and Methods

### 2.1. Materials and Chemicals

The chemicals used in this study included p-chloronitrobenzene, nickel sulphate (NiSO_4_·6H_2_O), ferrous sulphate (FeSO_4_·7H_2_O), sodium hydroxide (NaOH), hydrochloric acid (HCl) and sodium borohydride (NaBH_4_). All chemicals were of analytical grade and obtained from Sinopharm Chemical Reagent Co. Ltd. in Shanghai, China. Chromatographic methanol was acquired from Tianli Chemical Reagent Company (Tianjin, China). Deionized water was used in the preparation of the chemical solutions.

### 2.2. Preparation of Materials

The n-Fe/Ni was prepared by the liquid-phase reduction method. It involves the conventional reduction of Fe^2+^ with NaBH_4_ in a nitrogen (N_2_) atmosphere. Specifically, 40 mL of 1 M FeSO_4_ solution was put into a 500 mL three-necked round bottom flask. Then, 80 mL of 1 M NaBH_4_ solution was added to the FeSO_4_ solution dropwise (2 drop/s), under ambient temperature and mechanical stirring (150 rpm) to remove dissolved oxygen throughout the preparation process. N_2_ was purged into the three-necked round bottom flask during the synthesis. After the dripping, 3.2 mL of 1 M NiSO_4_ solution was added. The resulting mixture was mechanically stirred for 30 min to ensure uniform distribution of the Ni^2+^ on the surface of the nano-iron. The as-prepared material was filtered and washed with deionized water. It was later dried under vacuum at −30 °C for about 15 h in a freeze dryer and vacuum-packed and preserved in a refrigerator. The synthesized n-Fe/Ni was analyzed using an inductively coupled plasma emission spectrometer (Thermo Elemental, IRIS Advantage ER/5, Thermo Elemental Inc., Waltham, MA, USA). The result shows that the Ni:Fe mass ratio is 2.17:97.83.

### 2.3. Batch Experiments

Batch experiments for the coupling removal of the p-CNB and its reduction products (p-CAN and AN) were carried out in a 500 mL Erlenmeyer bottle. First, 0.5 g of freshly prepared n-Fe/Ni was added to 200 mL of the prepared p-CNB solution (10 mg/L, pH = 5–6) in a 500 mL Erlenmeyer bottle. The reaction was performed under ambient temperature and in a shaking incubator (200 rpm) to ensure adequate contact between the n-Fe/Ni and p-CNB solution. In order to determine the concentration of the p-CNB, p-CAN and AN, 2 mL of the supernatant was collected with a 5 mL syringe at pre-selected time intervals and filtered through a 0.45 µm nitrocellulose membrane. The batch experiments were performed in triplicate to ensure precision in the results obtained.

The filter liquors were analyzed by high-performance liquid chromatography (HPLC) (Ultimate 3000, Dionex, Sunnyvale, CA, USA) embedded with a C18 column (4.6 mm × 250 mm, 5 μm). The mobile phase of the HPLC contained chromatographic methanol and H_2_O at a volume ratio of 75:25 and operated at a flowrate of 1.0mL/min. The detector wavelengths were set at 270 nm for p-CNB, at 240 nm for p-CAN and at 230 nm for AN.

### 2.4. Material Characterizations

The morphological characteristics of n-Fe/Ni and iron oxides were analyzed using a transmission electron microscope (TEM) (Tecnai G2 F30, S-Twin, FEI, Hillsboro, OR, USA). The crystallinity of the materials was determined using an X-ray diffractometer (XRD) (D/MAX-2500, Science Corporation, Tokyo, Japan) operated at 45 kV and 250 mA with CuKα.

## 3. Results and Discussion

### 3.1. TEM Analyses

**Figure 1:**
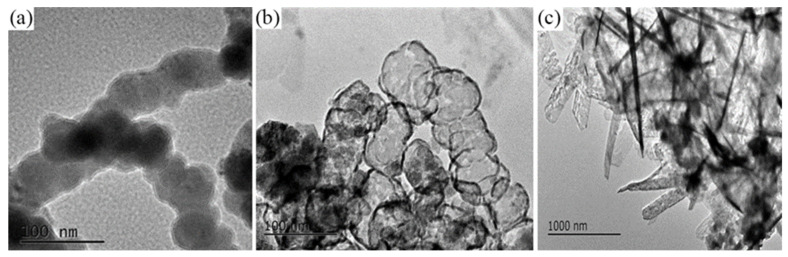
TEM images of Fe^0^ in n-Fe/Ni during reaction with p-CNB at different times: (**a**) 0 h; (**b**) 1 h; and (**c**) 8 h.

Figure 1 presents the TEM images of pristine and reacted Fe^0^ in n-Fe/Ni. The Fe^0^ nanoparticles in Figure 1a appear to be spherical and aggregated, thus forming chains [20,21]. This spherical and chain-like pattern has been attributed to the magnetic attraction of the iron [22,23]. After the 1 h reaction, the core of the Fe^0^ particles became hollowed and corroded (Figure 1b), indicating that the Fe^0^ in the inner core had almost completely reacted. Figure 1c displays the transformation of the spherical and chain-like pattern of the Fe^0^ nanoparticles into laths and needles, which are characteristic of lepidocrocite and goethite [24].

**Figure 2:**
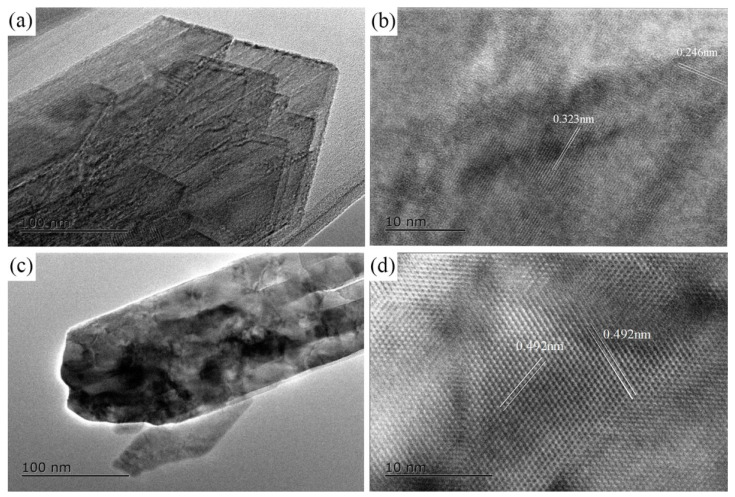
HR-TEM images of iron hydroxides formed by n-Fe/Ni reacted with p-CNB for 8 h. The image of lath iron hydroxide (**a**),the crystal plane spacings of lath iron hydroxide (**b**), the image of needle iron hydroxides (**c**) and the crystal plane spacings of needle iron hydroxide (**d**).

Figure 2 shows the HR-TEM images depicting the morphology and lattice fringes of the transformed products after the reaction of n-Fe/Ni with p-CNB for 8 h. In Figure 2a,c, the morphology of the iron hydroxides in the shape of a lath (Figure 2a) and needle (Figure 2c) can be clearly observed. This is consistent with the TEM observations (Figure 1c). The measured crystal plane spacings of lath iron hydroxideare 0.246 nm and 0.323 nm (Figure 2b), which are in line with the crystal planes (120) and (031) of rhombic structured lepidocrocite. The measured crystal plane spacing of needle-rod iron hydroxide is 0.492 nm (Figure 2d). This is consistent with the (020) crystal plane of rhombic goethite. HR-TEM analyses further proved that the iron hydroxides formed by n-Fe/Ni reaction with p-CNB for 8 h are lepidocrocite and goethite.

### 3.2. XRD Analyses

**Figure 3:**
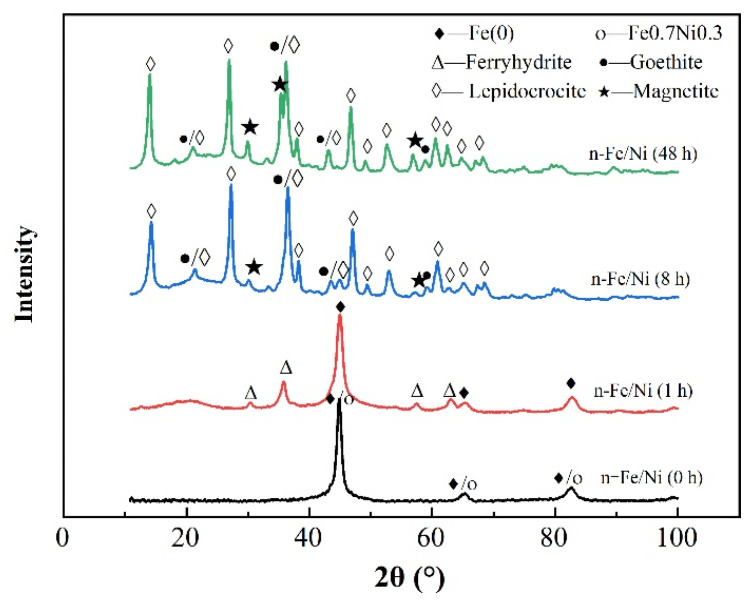
XRD patterns of n-Fe/Ni reacted with p-CNB at different times. Operating conditions: initial p-CNB concentration = 10 mg/L; n-Fe/Ni dose = 2.5 g/L; initial pH = 6.0, temperature = 25 ± 1 °C.

The XRD spectra of the n-Fe/Ni reacting with p-CNB at different times are shown in Figure 3. It is evident that the distinct peaks at 44.7°, 65.0° and 82.3°, which are characteristic of Fe^0^ and Fe0.7Ni0.3 (JCPDS No 06-0696), appeared in the pristine spectrum (0 h). This indicates the formation of both Fe^0^ and Fe0.7Ni0.3 alloy or solid solution before the reaction with p-CNB. At 1 h reaction time, new distinct peaks appeared at 30.4°, 35.8°, 57.5° and 62.8°. These peaks are belonged to the characteristic of ferrihydrite (Fe_5_O_3_(OH)_9_)( JCPDS No 29-0712). The peaks that depict the presence of Fe^0^ persisted until the 1 h reaction time. However, it illustrates that after 1 h of reacting the n-Fe/Ni with p-CNB, part of the Fe0 is converted to ferrihydrite. The mechanism of Fe^0^ transforming to ferrihydrite has been clearly explained in the literature [14]. At the 8 h reaction time, Fe^0^ and ferrihydrite are converted to lepidocrocite (γ-FeOOH—JCPDS No 44-1415) with characteristic peaks at 14.1°, 27.1°, 36.3°, 46.8° and 60.7°; to goethite (α-FeOOH—JCPDS No 29-0713) with characteristic peaks of 21.2°, 36.7°, 53.2° and 58.9°; and to magnetite (Fe_3_O_4_—JCPDS No 01-1111) with characteristic peaks at 30.1°, 35.5° and 57.2°. Considering that the Fe^0^ transforms into lepidocrocite, goethite and magnetite, it represents that the Fe^0^ converts into Fe^2+^ and then changes further to Fe^3+^ products with the help of H_2_O and the dissolved oxygen [14]. The XRD results are consistent with the HR-TEM results in terms of the reaction under 8 h. Although the characteristic peak of magnetite appears in the XRD pattern of n-Fe/Ni reaction for 8 h as well, its peak intensity is very weak, thus indicating that the content is small. This justifies the non-observance of magnetite in the HR-TEM results.

**Figure 4:**
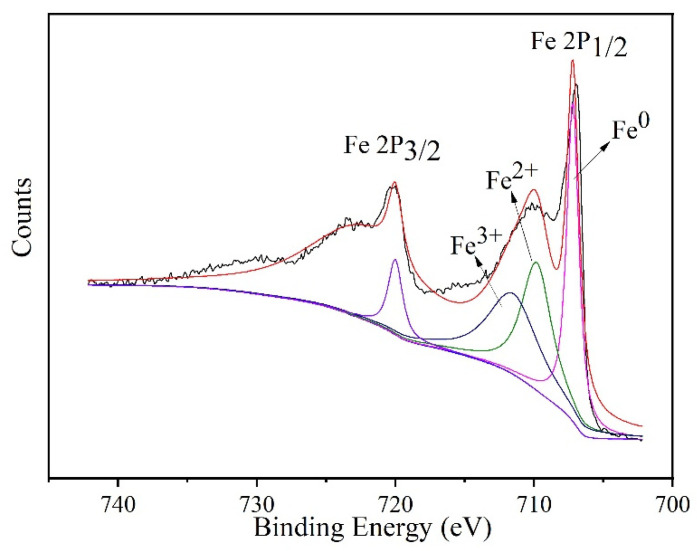
XPS analysis of n-Fe/Ni before the reaction with p-CNB.

Figure 4 shows the XPS spectrum of n-Fe/Ni before the reaction with p-CNB. It can be seen that the peak representing Fe^0^ at 706.7 eV is higher, which further confirms that the main content in n-Fe/Ni (0h) is Fe^0^. The intensity of the two peaks at 709.8 and 711.5 eV is relatively weak, indicating that only a small amount of iron exists in the forms of Fe^2+^ and Fe^3+^.

### 3.3. Effects of pH and Temperature on Coupling Removal of p-CNB, p-CAN and AN by n-Fe/Ni-FeOOH

#### 3.3.1. The Effect of pH Value on Removal Efficiency

**Figure 5:**
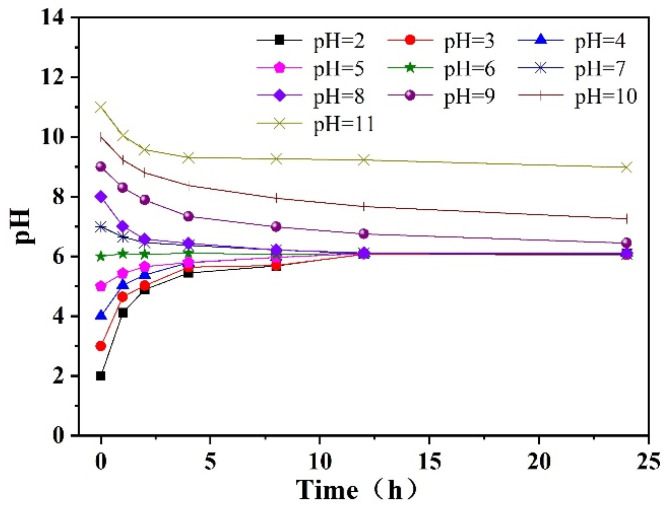
The variation in pH during the reaction of n-Fe/Ni with p-CNB.

A number of 200 mL p-CNB solutions with a concentration of 10 mg/L were taken. The pH of the solution was adjusted to 3, 4, 5, 6, 7, 8, 9, 10 and 11 with dilute HCl or NaOH; then, n-Fe/Ni was added to the solution at a dose of 2.5 g/L. The variation in pH during the reaction is shown in Figure 5. As can be seen from Figure 5, when the initial pH was ≤ 6, the pH of the solution increased with the extension of the reaction time and rose to about 6.0 after 28 h. When the initial pH of solution was 7, 8 and 9, the final pH of the solution decreased to between 6.1 and 6.4 afer 28 h. However, when the initial pH was 10 and 11, the pH of the solution decreased to 7.7 and 8.8 after 28 h of reaction, respectively. The reason may be that in the reaction between Fe^0^ and p-CNB, both H^+^ consumption and H^+^ generation occur (see Equations (1)–(6)). When initial pH was ≤ 6, H^+^ consumption was dominant; however, when initial pH was > 6, H^+^ generation prevailed. Since the pH of the reaction was stable at around 6 for all initial pH conditions (except for initial pH 10 and 11), no buffer solution was employed to maintain the pH of the solution during the reaction.

**Figure 6:**
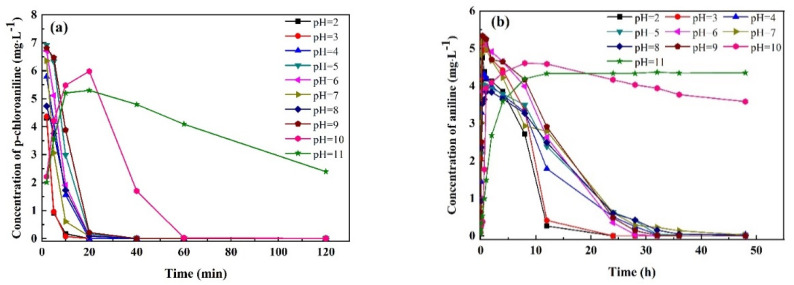
Effect of pH on the coupling removal of p-CAN (**a**) and AN (**b**) by n-Fe/Ni-FeOOH. Operating conditions: initial p-CNB concentration = 10 mg/L; n-Fe/Ni dose = 2.5 g/L; temperature = 25 ± 1 °C.

The influence of pH on the coupling removal of p-CNB, p-CAN and AN by n-Fe/Ni-FeOOH is shown in Figure 6. It is prudent to include that the p-CNB was quickly converted to its reduced form (p-CAN) within 2 min (except at pH 10 and 11) of reaction with the n-Fe/Ni. Therefore, the curve of the change in the concentration of p-CNB is not shown.

It is evident in Figure 6a that the reaction from pH 2 to 9 results in rapid dechlorination of the p-CAN within 20 min. This may be because the weak acid–weak base condition is favorable for n-Fe corrosion, thus providing sufficient H_2_ and further producing more H* under the action of Ni [25]. On the other hand, when pH = 2–9, the passivated layer on the n-Fe surface will be dissolved and thinned to some extent. Therefore, the activity of Fe^0^ at the reaction site can be better developed. When pH > 9, the dechlorination efficiency of n-Fe/Ni for p-CAN becomes slower, eventually dechlorinating by 60 min and remaining constant until 120 min. When pH = 11, the dechlorination performance of n-Fe/Ni reduces drastically and the dechlorination of the p-CAN is still not completed within 120 min (Figure 6a). The reason is that Fe (OH)_2_ and Fe (OH)_3_ precipitated on the n-Fe/Ni nanoparticles to cover the reaction site of Fe^0^ under alkaline conditions, making it difficult for the Fe^0^ to exert its activity [26,27,28].

In Figure 6b, the adsorption rate of the AN by FeOOH, which is formed after the transformation of the Fe^0^ reacted for 8 h, is relatively fast when the pH is 2 and 3. Complete adsorption of the AN by the FeOOH occurs within 24 h of reaction. When the pH is between 4 and 9, the adsorption rate of the AN by FeOOH becomes slow, yet it is eventually completely adsorbed within 36 h of the reaction. However, when the pH is 10, the adsorption rate of the AN by FeOOH is only 21.1% for 48 h. At pH = 11, FeOOH completely loses its adsorption capacity for AN since the concentration of the AN does not decrease between 10 and 48 h. Therefore, only in acidic or slightly alkaline environments can FeOOH have a good adsorption capacity for AN [29]. Furthermore, the lower the pH, the faster the adsorption rate of AN by FeOOH [30].

#### 3.3.2. The Effect of Temperature on Removal Efficiency

**Figure 7:**
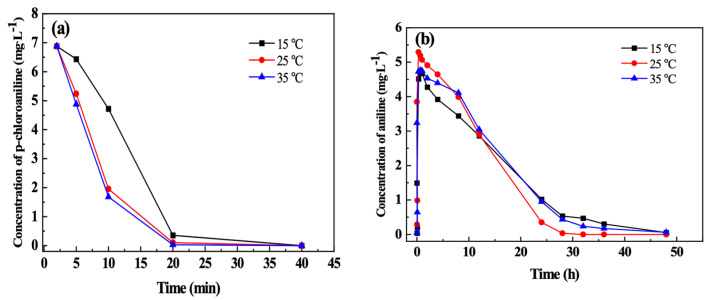
Effect of temperature on the coupling removal of p-CAN (**a**) and AN (**b**) by n-Fe/Ni-FeOOH. Operating conditions: initial p-CNB concentration = 10 mg/L; n-Fe/Ni dose = 2.5 g/L; initial pH = 6.0.

Figure 7 indicates the effect of temperature on the coupling removal of p-CAN and AN by n-Fe/Ni-FeOOH. It can be observed from Figure 7a that there is a positive proportional relationship between temperature and the dechlorination of p-CAN by n-Fe/Ni. The reason is that the mass transfer rate in the reaction system accelerates with the increase in temperature and promotes dechlorination [31]. In addition, the changes in temperature lead to changes in the concentration of the dissolved oxygen (Table 1). Specifically, there is a negative correlation between the temperature and the solubility of oxygen in water, which is not conducive to the formation of the passivation layer on the n-Fe/Ni surface. Therefore, the active site of the Fe^0^ on the n-Fe/Ni surface is fully utilized. Although the dechlorination rates are slightly varying with different temperatures, p-CAN is completely dechlorinated within 40 min by the n-Fe/Ni at 15, 25 and 35 °C. The test results illustrate that temperature has little influence on the dechlorination of p-CAN.

Figure 7b demonstrates that AN can be adsorbed by the FeOOH formed after the reaction between n-Fe/Ni and p-CNB at different temperatures. However, the adsorption rate of the AN by FeOOH is much faster at 25 °C than the other two scenarios. The reason is that Fe^0^ can be converted into the more crystalline FeOOH at such temperatures [32,33,34].

### 3.4. Process and Mechanism of Coupling Removal of p-CNB, p-CAN and AN by n-Fe/Ni-FeOOH

**Figure 8:**
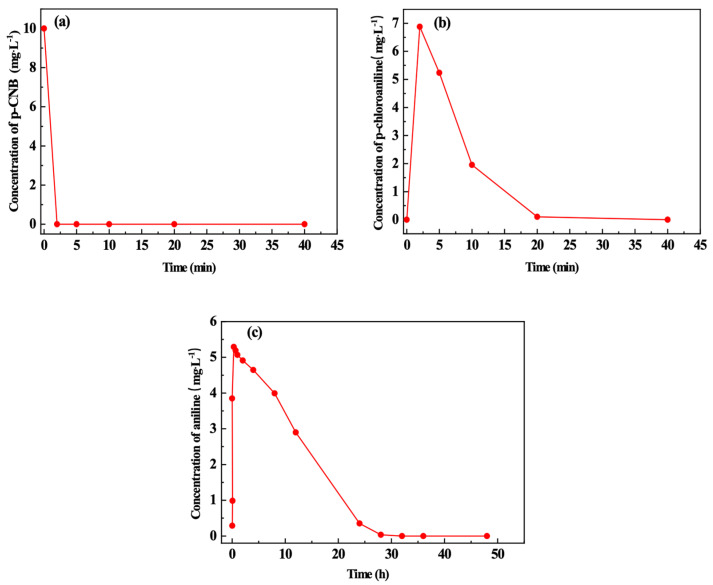
Variation in the concentration of p-CNB (**a**), p-CAN (**b**) and AN (**c**) over time. Operating conditions: initial p-CNB concentration = 10 mg/L; n-Fe/Ni dose = 2.5 g/L; initial pH = 6.0.

Figure 8 shows the variation in the concentration of p-CNB, p-CAN and AN over time. In Figure 8a, p-CNB is completely removed within 2 min after n-Fe/Ni is added. This is because Fe^0^, as an electron donor, reduces –NO_2_ on p-CNB into the –NH_2_ group (Equation (1)), so that p-CNB is converted to p-CAN. Meanwhile, Ni and Fe^0^ form the Fe-Ni galvanic cell, which accelerates the electron loss in Fe^0^ corrosion and further hastens the electron transfer rate. The concentration of p-CAN reaches its peak (6.8 mg/L) at 2 min and then decreases to close to 0 at 20 min (Figure 8b). In the meantime, Figure 8c indicates that the concentration of AN reaches its peak (5.2 mg/L) at 20 min. This demonstrates that p-CAN has been completely converted to AN.

In the dechlorination process, only Fe^0^ cannot provide electrons to achieve dichlorination [18]. Fe^0^ reacts with water to form H_2_ (Equation (2)). And then H_2_ is converted by the catalyst Ni into active hydrogen atom (H*) (Equation (3)) [35,36,37]. Immediately, H* acts on the C-Cl bond of the benzene ring to hydrochlorinate p-CAN into AN [38,39].The removed chlorine atom gains electrons to become the chloride ion (Equation (4)).

When p-CNB is nitro-reduced and dechlorinated by n-Fe/Ni, Fe^0^ reacts with the dissolved oxygen and water to transform into FeOOH (Equation (5)), which can form a complex with AN to adsorb AN (Equation (6)) [40,41]. As can be seen from Figure 8c, AN can be completely adsorbed by FeOOH after 28 h and the adsorption rate of AN is fastest between 8 and 28 h.

(1) $${\mathrm{Fe}}^{0}+Cl─R─NO_{2}+6H^{+}\to Fe^{2+}+Cl─R─NH_{2}+2H_{2}O$$

(2) $$Fe^{0}+H_{2}O\to Fe^{2+}+H_{2}+2OH^{-}$$

(3) $$H_{2}\overset{Ni}{\to }2H^{*}$$

(4) $$H^{*}+Cl─R─NH_{2}+e^{-}\to R^{\prime }─NH_{2}+Cl^{-}$$

(5) $$2Fe^{2+}+1/2O_{2}+3H_{2}O\to 2FeOOH+4H^{+}$$

(6) $$\equiv FeOOH+R^{\prime }─NH_{2}\to FeOOH─H_{2}N─R^{\prime }$$

Considering that during the reaction of n-Fe/Ni with p-CNB, Ni deposited on the surface of Fe^0^ may react with dissolved oxygen to form Ni^2+^ and enter the solution, the concentration of Ni^2+^ in the solution after the reaction for 48 h was determined by an inductively coupled plasma emission spectrometer. The concentration of Ni^2+^ was 0.073 mg/L. Therefore, the heavy metal Ni in n-Fe/Ni will not cause secondary pollution in the coupling removal of p-CNB, p-CAN and AN by n-Fe/Ni-FeOOH.

**Figure 9:**
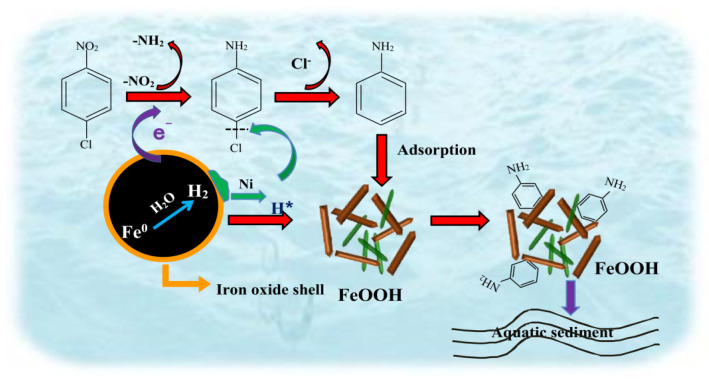
Schematic representation of the process and mechanism of the coupling removal of p-CNB, p-CAN and AN by n-Fe/Ni-FeOOH.

Based on these analyses, a schematic representation of the process and mechanism of the coupling removal of p-CNB, p-CAN and AN by n-Fe/Ni-FeOOH is shown in Figure 9.

## 4. Conclusions

Coupling removal of p-CNB and its nitro-reduction products (p-CAN) and dechlorination products (AN) by n-Fe/Ni-FeOOH were investigated. XRD and TEM results show that after 8 h of reaction, the transformation products of Fe^0^ are mainly lath-like lepidocrocite (γ-FeOOH) and needle-like goethite (α-FeOOH). The dechlorination efficiency of n-Fe/Ni for p-CAN and the adsorption rate of FeOOH for AN are both high, with pH between 2.0 and 9.0, while the efficiency is poor at pH > 9.0. The dechlorination of p-CAN and the adsorption of AN are less affected by temperature. The process and mechanism of n-Fe/Ni-FeOOH coupling removal of p-CNB and its reduction products are as follows: Fe^0^ in n-Fe/Ni provides electrons to reduce p-CNB to p-CAN and then dechlorinates p-CAN to AN under the catalysis of Ni. Meanwhile, it is oxidized to FeOOH by dissolved O_2_ and H_2_O. AN is then adsorbed by FeOOH, so that p-CNB, p-CAN and AN are completely removed.

## Figures and Tables

**Table 1:** Solubility of oxygen in water at different temperatures.

Temperature (°C)	Solubility (mg·L^−1^)	Temperature (°C)	Solubility (mg·L^−1^)
0	14.64	20	9.08
5	13.15	25	8.28
10 15	10.24 9.86	30 35	7.56 6.95

* Dissolved oxygen in water at different temperatures was measured by a portable dissolved oxygen meter (LEICI JPSJ-606L, INESA Co.,Ltd., Shanghai, China).

## Data Availability

All relevant data presented in the article are stored according to institutional requirements and as such are not available online. However, all data used in this manuscript can be made available upon request to the authors.
